# The role of ‘*accompagnement*’ in the end-of-life debate in France: from solidarity to autonomy

**DOI:** 10.1007/s11017-016-9389-1

**Published:** 2016-12-03

**Authors:** Marie Gaille, Ruth Horn

**Affiliations:** 1 Laboratoire SPHERE (UMR 7219), University of Paris Diderot—CNRS, bâtiment Condorcet, case 7093, 5 rue Thomas Mann, 75205 Paris Cedex 13, France; 2 The Ethox Centre, Nuffield Department of Population Health, University of Oxford, Old Road Campus, Oxford, OX3 7LF UK

**Keywords:** Autonomy, Solidarity, End of life, International bioethics, Palliative care, Accompagnement, France

## Abstract

This article traces the way autonomy has become a recognised value in health care in France. In a country that based its social fundamentals on the very idea of solidarity for many years, autonomy has long been considered a foreign ‘Anglo-American principle’. Taking the example of the end-of-life debate, the article shows, however, how the use of the French term ‘*accompagnement*’ allowed autonomy to be redefined and to be associated with the concept of solidarity. Exploring the arguments used over the past 25 years in professional guidelines, parliamentary reports, ethics committee reports, and legal texts, the authors describe the shift that took place in public and legal discourses on end-of-life care. The analysis demonstrates how the scope of autonomy has been limited by other social values, such as the protection of the dying person, in order to become an accepted social value in its own right. The example of the French end-of-life debate shows that depending on how the concept of autonomy is adapted and applied in a specific context, it can be compatible with the idea of solidarity. Such compatibility has been challenged previously in the international bioethics debate. By demonstrating the possibility of combining autonomy and solidarity, this article makes an important contribution to the international bioethics debate and to the dialogue between countries that are often perceived as significantly different.

## Introduction

Continental European and Anglo-Saxon approaches to health care are often associated with two seemingly opposed conceptions of care: in continental European countries, solidarity appears to be the core value of health care systems, whereas a rights-based, individualistic conception seems to prevail in Anglo-Saxon countries. The prominence of solidarity in the continental approach is often considered a detriment to respect for patient autonomy and seen as permitting paternalistic relationships. We challenge this opposition, first, because both autonomy and solidarity are notions that can have various meanings and, second, because the notion of solidarity can make a meaningful contribution to the theoretical debate on autonomy and vice versa. In this article, we use the end-of-life debate in France as a case-study of how autonomy and solidarity can be combined. We show that this combination allows one to see the individual as a related being, rather than an atomistic subject.

Since its emergence in the late 1970s, the end-of-life debate in France has become more intense and radical.^1^ From the 1990s onwards, this development was demonstrated by the increase in the number of end-of-life cases reported in the media, and by more frequent discussion of the issues in professional, political, and legal medical discourses [1–6]. This change is of particular interest to ethical and political analysis. As some philosophers and social scientists have observed, controversies that arise at the patient’s bedside and in the legal and political context reflect values and principles that are dominant in a specific context [7–10].

In the past 25 years, the French end-of-life debate has undergone a significant shift from a solidarity or community orientation to a more autonomy-based approach. As we will show, the notion of autonomy was initially considered to be a foreign ‘Anglo-American’ concept and was regarded with scepticism. Autonomy was presented as an antonym to ‘solidarity’, an idea which was taken for granted and could easily be used to justify end-of-life practices. Increasingly, however, the notion of autonomy began to emerge in French medical ethics and in the legal discourse. Ultimately, it became an important and socially accepted value that was no longer questioned. Currently, an attempt is being made to ‘reconcile’ the notions of autonomy and solidarity. We will illustrate this attempt through the analysis of the use of the French term *‘accompagnement*’ which could be best translated as ‘support’ or ‘presence’. This term is used in ordinary French language, but has gained a normative connotation in medical practice.

In this article, we discuss the use of and the meaning attributed to the notions of solidarity and autonomy in the French end-of-life debate. Our aim is to better understand this specific debate in order to highlight its interest for the international bioethics debate. Values, norms, and principles are often used and defined in many different ways. As Diego Gracia has noted, the notion of autonomy has ‘many faces’ [11]. It is the same for the notion of solidarity [12, 13]. We will discuss whether and how their meanings have been adapted to meet goals and needs that are specific to the French debate.

Our analysis focuses on public and legal discourses. From a methodological point of view, we will examine a series of texts that are exemplary for the public and legal debate, such as professional guidelines, parliamentary reports, ethics committee reports, and legal texts. Our examination aims to identify (1) the perceived ethical problems associated with the end of life in France; (2) the way medical practice and social attitudes have developed in the course of the debate; (3) the way moral and legal legitimacy of end-of-life practices is achieved. We will first show how autonomy and solidarity became part of the moral reflection about medical decision making in France (first section). We will then examine how the notion of autonomy has found its place in the debate about end-of-life care up to the present (second section). Finally, we will present an analysis of the moral scope and limits of the shift towards a combination of solidarity and autonomy in the French end-of-life debate (third section).

## Solidarity and autonomy in the French health care debate

Both autonomy and solidarity are key notions in the French end-of-life debate, as well as in other debates about ‘good’ medical practice, e.g., regarding assisted reproduction. The importance of these two notions in the end-of-life debate is the result of a complex history. Tracing back this development allows us to understand how this debate took shape, and which normative arguments were employed. It also highlights the specific meaning and use of the notions of autonomy and solidarity in the end-of-life debate.

As a first step, we discuss the meaning of solidarity. Despite the association that can be made with the idea of *obligatio in solidum* in Roman law or with that of the Republican principle of *concordia* or the Christian *fraternitas*, ‘solidarity’ is a modern term. The term ‘solidarity’ appeared in dictionaries only at the end of the 18th century and was first used in a legal context in Europe. Following the French Revolution, the term was employed in political theories in order to combine the principle of individual liberty with the idea of social cohesion and community [14]. This idea was taken up and developed by the founding fathers of sociology, Auguste Comte and Émile Durkheim. Despite the fact that the term solidarity emerged in the context of the French Revolution, it spread across Europe and took on different meanings [15, 16]. The multiple meanings associated with solidarity may have contributed to its negative connotation as a ‘wishy-washy concept’ [12] or a purely ‘rhetorical idea’ that is interchangeable with other terms such as ‘fraternity’, ‘brotherhood’, ‘community’, or ‘unity’ [14, 17].

In France, however, the notion of solidarity always occupied a prominent place, notably in the development of its health care system. Already prior to the French Revolution, hospitals were established to receive poor and sick people, old persons, young orphans, or abandoned children. During the Revolution, this policy, based on the principle of solidarity, was reinforced with the introduction of a publicly-funded system, the ‘*assistance publique*’. From the late 19th century onwards, until World War II, several laws enabled access to medical care in public hospitals for more and more types of sick people. In 1945, the establishment of the social security system gave universal access to hospital care. Based on the principle of ‘solidarity’, it was deemed that every French citizen should enjoy equal access to the health care system, regardless of their income and origin [18–23].

In the French context, solidarity has two meanings: firstly, it implies the public organisation of the health care system, as opposed to an organisation exclusively based on private structures and charity funds; secondly, it suggests a bond between the members of society, who must help each other through hardship. It has a normative meaning and describes a type of ‘non-instrumental cooperation’ [12]. To a certain extent, during the course of French history, ‘solidarity’ became part of daily language, a term that needed no further definition. Yet, as we will show through the example of the end-of-life debate, its meaning could change depending on circumstances and political or social motivations.

The emergence of the notion of autonomy, in terms of patient autonomy, relates to recent history. In France, as well as in other Western societies, patient autonomy has three meanings [24]. It was first recognised as a goal of medicine which aimed to restore bodily functions. Medical teams still speak today of ‘functional autonomy’ in relation to the physical capacities of the patient. Then, following the increasing prevalence of chronic disease due to early detection and improved treatment (e.g., the discovery of insulin in 1921), the idea of patient autonomy also took on the sense of patient self-management. Finally, since the Nuremberg trials in 1947, respect for patient autonomy has become a prevailing, ethical principle regulating human experimentation and medical decisions at the bedside. As Robert M. Sade pointed out, ‘one of the earliest projects of bioethics was to promote patient autonomy in an effort to counter the paternalistic decision-making by physicians that was so deeply embedded in medical practice before the 1960s’ [25]. In the history of bioethics, this idea has at least two dimensions: patient privacy and self-determination.

In France, the criticism of the paternalistic model developed in the 1970s within philosophy and the social sciences, noticeably in the works of Georges Canguilhem and Ivan Illich; it was reinforced by the early translations of Ervin Goffman and Eliot Freidson [26]. The rejection of the paternalistic approach was triggered by the development of the HIV patient movement. These patients did not want to be seen as vulnerable patients any more, but as competent persons able to make decisions about their own lives and their participation in research protocols [27–30]. From the 1980s, this criticism of the paternalistic model was also based on various issues extensively covered by the daily press, such as the contaminated blood scandal from the 1980s to the 1990s or cases of chemical pollution [31–33]. These events had two consequences: first, ‘trust’ in medicine and in the public health system significantly decreased; second, from the 1980s on, French citizens were very concerned about reforming the law about medical decision-making. Onora O’Neill’s analysis of trust and autonomy shows a good grasp of the issues at stake in this debate [34].

However, the criticism of the paternalistic approach and the shift towards a ‘democracy in health care’ (*démocratie sanitaire*) did not entail a consensual acceptance of the idea of patient autonomy in France. Patient self-determination was regarded with suspicion, and still is to a certain extent. Autonomy was not necessarily considered to be a good in itself and the protection of the ‘vulnerable’ patient seemed to be more important.

Today, we observe that the French health care system is evolving towards a patient-centred approach and is beginning to take on board the idea of shared decision-making. Special attention is paid to the issue of patient autonomy in more cases in which patients appear unable to make decisions for themselves [35–38]. However, many studies stress the limits of this development in French medical practice and some scholars object that the new ‘patient-centredness’ is only ‘rhetorical’ [26, 39–42].

The end-of-life debate plays a key role in the discussion about the understanding and acceptance of autonomy in French health care. In the following section, we will focus on how ambiguity with regard to patient autonomy influences public documents and law.

## The French end-of-life debate and its ‘struggle’ with autonomy

Since the beginning of the end-of-life debate in France in the 1980s, professional guidelines, committee reports, and laws provide evidence of the difficulty of employing the notion of autonomy. Although paternalism has been increasingly criticised in the public and professional discourse, respect for autonomy is still finding a place in the debate. In addition to a persisting paternalism, we also observe tensions between a certain understanding of patient autonomy (in terms of self-determination) and the arguments used by proponents of palliative care in order to challenge a patient’s request to die.

Palliative care developed in France from the 1980s onwards, but, in contrast to other countries, the expression ‘hospice movement’ never became established there. The expression ‘palliative care and *accompagnement*’ was used instead [5]. Along with the development of palliative care in France, the term ‘*accompagnement*’ gradually gained in importance in the end-of-life debate. Proponents of palliative care argued that requests for the right to die do not necessarily express autonomous wishes, but rather the fear of dying alone and abandoned by society. They claimed that patients at the end of life needed to be supported by their doctors and carers even when no further treatment is available [5]. The focus of the palliative care movement was, and still is today, on support of the dying patients and their loved ones.

In a response to an increasingly vehement and polarised right-to-die debate, the French government opted to focus on the development of palliative care in the public health care system. In 1986, a regulatory text, the *Circulaire Laroque*, stated that patients at the end of life should be taken in charge by palliative care teams, who would support the person in their final days and respond to their physical, psychological, spiritual, and social needs [43]. A few years later, a law was passed in 1991 requiring the provision of palliative care and *accompagnement* by the public health care system [44]. Several parliamentary reports emphasised, however, the lack of structures guaranteeing the support of end-of-life patients and a subsequent law was passed in 1999 [45]. This law established a right to palliative care and *accompagnement*, and a right to oppose medical examinations and therapeutic interventions. Physicians, however, were not legally required to respect such an opposition.

Whereas the notion of *accompagnement* emphasises that the dying patient is a person with individual needs and values, the focus on autonomy could not serve to justify respect for patients’ wishes in the French debate. The notion of *accompagnement* implies patients’ embeddedness in society, rather than putting the focus on their individuality: medical teams and society at large contribute to an active and supportive presence in the last moments of life.

During this time, the notion of solidarity was rarely employed in the debate. The notion was mainly understood to be relevant to political and legal debates, and although it was presented as a core value of the French health care system, it was not part of the usual categories of ethical reflection about medical decision-making at the bedside. However, it was used once to argue in favour of a law on ‘euthanasia’ in exceptional cases; this was in a report of the French national ethics committee (CCNE) in 2000 [46]. In this report, the CCNE discusses the challenges of end-of-life care in the light of technical developments and a changing society. The report emphasises the importance of supporting patients in the final stage of life and of taking into account their individual wishes, beliefs, and needs, rather than insisting on futile treatment. The committee presents palliative care as an answer to difficult end-of-life situations. However, the CCNE admits that in the exceptional case of unmanageable pain, or where patients are dependent on life support and deprived of their relational capacities, their wish to die should be granted. According to the committee, such situations of distress require society’s ‘human solidarity and compassion’ and justify the ‘exceptional practice of euthanasia’. The committee’s report was widely debated in French society, yet it had no impact on legislation.

Nevertheless, at this moment, patient rights were increasingly under discussion in France and became the subject of legal reforms. Normative reflection entered a new phase. While some scholars still argued that autonomy was an inappropriate ethical principle on which to base the care of ‘vulnerable patients’ [47], others argued that autonomy could no longer be ignored.

Despite the ongoing ambivalence towards autonomy, in 2002, a law was passed to enforce the rights of patients, e.g., their right to refuse treatment [48]. However, in 2003, the case of Vincent Humbert, a paraplegic patient who claimed his right to die, highlighted the uncertainties of physicians regarding the legality of withdrawing life-supporting treatment such as clinically-assisted hydration and nutrition [5]. This case attracted much media attention and generated, among other things, an important parliamentary report in 2004 [49] and a report by the CCNE in 2005 [50]. Both reports stressed the need to improve the support of end-of-life patients and to reassure physicians of the legality of withdrawing even life-sustaining treatment. The CCNE’s report presents an ambiguous argument regarding refusal of treatment by a patient. Although the committee’s aim was to define a solid ethical argument justifying treatment refusal, it tended to emphasise such situations in which refusals should be challenged: e.g., cases of cognitively impaired patients, addicted patients, or patients who suffer from psychiatric disorders or emotional distress. Finally, using the same arguments as palliative care supporters, the CCNE report states that refusal of treatment sometimes expresses a call for help and should be understood as such by the medical team.

Following the discussions around the Humbert case, a new law was passed in 2005 [51]. This law explicitly introduced the right to refuse ‘every’ treatment, including clinically-assisted nutrition and hydration, and defined the physician’s right to withdraw treatment at the end of life. This law clearly defined physicians’ ‘right to let a patient die’ and their obligation to maintain the quality of life and dignity of the dying person. The law also promoted the development of palliative care. Although the law of 2005 aimed to strengthen the respect for patient autonomy, it was also evidence of ambivalence towards that autonomy. Firstly, it was only applicable to ‘competent’ patients. Secondly, it stated that the physician should ‘do all that is possible’ in order to persuade the patient to continue the treatment, when the refusal endangered the patient’s life, and the patient was required to repeat their wish to discontinue treatment. As the legal scholar Dominique Thouvenin pointed out, in a clause that establishes a patient’s subjective right, in other words, that defines the patient as the right-holder, such limitation appears to be a paradox [41].

This paradox, or the struggle to respect patient autonomy, also showed in the value attributed to advance directives to refuse treatment when patient are no longer able to express their wishes. Until 2016, advance directives only had advisory value in French law. The law of 2005 stated that the physician may take these documents into account but is not obliged to respect them. As Horn has shown, advance directives have long been considered as individualistic ‘Anglo-Saxon inventions’ that have no place in France, ‘a country with social values’ [6]. The parliamentary report of 2004 argued that according binding value to advance directives could weaken both the family’s and the physician’s sense of responsibility for the patient’s physical welfare, as well as their solidarity with the vulnerable person [49].

From the very beginning, the 2005 law was discussed as a provisional legal framework. Several cases which were discussed in the media showed its limitations in addressing the following: patients’ needs for effective pain management at the end of life (case of Hervé Pierra); their requests for a self-determined death (case of Chantal Sébire); and the procedures required to withdraw treatment at the end of life, in the case of conflict of opinion (case of Vincent Lambert). A parliamentary report of 2008 confirmed the shortcomings of the law and its application in medical practice [52].

The French president François Hollande set up a consultation in order to review the law. This resulted in a new report being published by a parliamentary committee in 2012 [53]. This report, entitled, ‘Reflections on the End of Life from a Perspective of Solidarity’ (*Penser Solidairement la Fin de Vie*) made two suggestions, in particular, on how to improve the law on end-of-life treatment. Firstly, the report suggested granting greater value to advance directives, i.e., strengthening patient autonomy. Secondly, and as a ‘French solution’ to problems regarding the respect for patient wishes, the report suggested improving the support (*accompagnement*) of dying patients. The report recommended that palliative care should be provided from the very moment the patient is diagnosed with a serious illness, and continuous deep sedation should be offered at the request of patients deciding to discontinue life-sustaining treatment, e.g., clinically-assisted nutrition and hydration.

The report of 2012 illustrates a new and growing use of the term ‘*accompagnement*’ in the sense of promoting both solidarity and autonomy. This term, so long associated with palliative care, expresses the duty of society towards dying patients and their loved ones, a duty which implies a confrontation with death, a fight against the feeling of abandonment and loneliness, and finally, equal access to palliative care. ‘*Accompagnement*’ has long been an expression of solidarity. The report of 2012 also refers to solidarity but includes respect for autonomy as a new element in the *accompagnement* of dying patients. The word ‘*accompagnement*’ is not precisely defined in this report. However, the text indicates that *accompagnement* had become a central theme in the reflection about end-of-life care. According to the report, *accompagnement* is considered necessary to coping with the fear of abandonment and the anxiety of dying felt by many patients. It is also seen as an important element in encouraging people to openly discuss end-of-life issues. At a political level, social inequalities in access to end-of-life care and support for proxies must also be dealt with. Finally, *accompagnement* is also associated with an attitude of respect for patient wishes. This aspect relates *accompagnement* to patient autonomy since the report rejects decisions that do not take into account the patient’s view.

For these reasons, ‘*accompagnement*’ appears in the report as the leading principle of a society that takes care of dying patients, and solidarity is rendered as the social will to respect patient autonomy. The importance of respect for patient autonomy is no longer questioned. Two years later, in 2014, the CCNE report on the public debate about the end of life made a similar use of the notion of *accompagnement* [54] and discussed the same association between autonomy and solidarity. It argues in favour of a *‘véritable accompagnement humain’* aiming to offer both care and cure and to address the fear of abandonment, pain, and social inequalities in access to care. Above all, this *accompagnement* consists in listening to patients’ wishes, including their wish to stop treatment. Once again, the respect for patient autonomy is associated with the concern to guarantee social solidarity in the last stage of life.

As a result of further debates and another parliamentary report in 2015 [56], a new law on end of life issues came into force early in 2016. The ‘law creating new rights in favour of patients and persons at the end of life’ recognises the right to continuous deep sedation and grants binding value to advance directives [57]. A closer analysis of this law shows, however, that although respect for autonomy is now considered an important value, its implementation is not straightforward. The same paragraph (L.1111-11, Public Health Code) that states that advance directives are binding for the physician also mentions that the physician may challenge such a directive if it appears ‘clearly inappropriate’ to the medical situation of the patient. If a physician refuses to implement an advance directive, this decision is re-examined in a so-called collegial procedure (i.e., the treating physician has to consult other physicians). By contrast, with other nations such as Germany or England, the physician is not required to discuss the advance directive with the patient’s family and loved ones in order to establish the presumed wishes of the patient. As Thouvenin points out, the decision of whether to apply an advance directive remains a medical decision. According to French law, only physicians are able to validate the authenticity or appropriateness of a previously expressed patient wish [58]. Although French law has increasingly strengthened the right of competent patients to make their own decisions, this is not the case for patients who are no longer competent to express their will, even if they have a previously written advanced directive.

Our analysis of 25 years of French end-of-life debate shows that although patient autonomy has gradually been established as an important value, it is still not easily implemented in the French context. However, the situation has not remained the same in the course of the debate; a way to combine autonomy and solidarity has been found that makes it possible to argue in favour of respect for patient autonomy. Today, in France, those who advocate respect for patient autonomy are sometimes doing so for the sake of individual self-determination. Such respect is also justified on the basis of an autonomy that is conceived as a shared collective value. In this perspective, respect for patient wishes is seen as a leading principle in end-of-life care, not because of a rights-based individualistic conception, but because of the social duty to address the needs of dying patients. It is thus on the basis of social solidarity that the respect for patient autonomy has become legitimate in end-of-life care. As we will show in the following section, the support of terminally ill patients expresses this new combination between solidarity and autonomy. We will also examine the limits of patient self-determination, and finally, develop our reflection on the implications of the attempt to bring together autonomy and solidarity in order to define ‘good’ end-of-life care.

## Scope and limits of associating autonomy with solidarity

From the 1980s on, the French debate regarding end-of-life care was intense and heated. Gradually, this debate became more nuanced and its focus shifted from the right to die to more practical questions about ‘good’ end-of-life care. As a response to ‘the painful ordeal’ caused by futile treatment at the end of life [46], palliative care was integrated into hospital settings. As mentioned, in the first instance, the term ‘*accompagnement*’ was strongly associated with palliative care and the support of the dying person surrounded not only by professionals, but also by their loved ones. In a first phase, ‘*accompagnement*’ implied respect for the patient as a person. However, it did not refer to the notion of autonomy. Patients had no right to decide about their end-of-life care. In retrospect, this social and legal pattern may be considered paternalistic.

In this first phase, the term ‘autonomy’ was used to defend the right to ‘reclaim one’s own death’ [46], a request considered socially unacceptable in the policy and legal documents we have presented. Hence, autonomy could not be employed on its own as a principle to justify respect for patient wishes. Rather, the request to choose one’s own death could only be taken into account if solidarity was put forward as the leading moral value in end-of-life care. When arguing for euthanasia, the right to choose the moment of one’s death under ‘exceptional circumstances’, the CCNE report of 2000 emphasised society’s duty to show solidarity with the suffering person [46]. By referring to solidarity, a widely accepted value in French society and health care, this 2000 report intended to find an ethical justification for the highly controversial act of euthanasia based on a patient’s wish. It, however, remained an isolated view.

From 2005, in the aftermath of the Humbert case, *accompagnement* was increasingly presented as an important part of medical practice, particularly where active treatment was foregone. *Accompagnement* was no longer exclusively linked to the provision of care for dying patients but also applied to all patients who refused treatment, even if they were not in the last stage of their life. Henceforth, *accompagnement* represented respect for patient wishes, beliefs, and values. Physicians were now expected to provide continuous support for patients even if they refuse further treatment. The term *accompagnement* began to embrace more than just palliative care, and was extended to care in general, whether it was medical, spiritual, or social. In 2009, the National Institute for Prevention and Health Care Education (NPES) defined *accompagnement* as the ‘human’ capacity to listen to and engage with the patient, and to show consideration for her or him. Increasingly, the patient became the centre of attention as a person with her or his own beliefs and values [59]. This vision of *accompagnement* strengthened the position of the patient at the end of life only up to a certain extent. It expressed an ethical concern about professional attitudes towards dying patients rather than actually focusing on patient self-determination.

From 2012, the debate focused on the question of whether patient wishes could be challenged at all. In this context, the support of patients was perceived as part of a broader social commitment not to abandon the dying person, while respecting their wishes, rather than submitting them to paternalistic medicine. *Accompagnement* was understood as a way of expressing solidarity, both at an individual and a social level.

The term ‘*accompagnement*’ is worth commenting upon: it is not an ethical or philosophical notion, but a term used in ordinary language and daily life, and easily understood by everyone. It has a positive meaning and describes a way of ‘being with others’, a willingness to ‘stand by each other’ [12]. A ‘companion’ designates a person a person can count on, who respects her, shares her interests and does not try to dominate her. It is widely used to describe good professional practice, not only in medical contexts, but also in the broader context of social community care and education. As such, the term is used to emphasise both respect for another person’s wishes, beliefs, and needs, and the support provided by being ‘at their side’.

In the strongly polarised French end-of-life debate, the use of the term ‘*accompagnement*’ is not incidental. Facilitated by the ordinariness of the term in French language and its broad acceptance in French society, it allows its users to describe a viable combination of autonomy and solidarity. It is not a theoretical justification of this combination, but it is an effective rhetorical tool for conveying the combination in the public debate, both at an ethical and legal level. On the one hand, *accompagnement* allows acceptance of patient autonomy within some limits, as we will see below. In legal terms, since February 2016, this means that patients who are able to express their will are entitled to receive every necessary intervention to alleviate their pain. Patients may also refuse medical treatment, including nutrition and hydration. Finally, the law sets up a procedure for continuous deep sedation when the suffering of the patient is considered unbearable and the patient is in her last stage of life. On the other hand, the use of the word ‘*accompagnement*’ is also a way to acknowledge the role of medicine and society as a whole: patients are those who are ‘accompanied’ in the sense of supported; and medicine and society are their ‘companions’ who provide the support. They are accounted responsible for creating the necessary conditions for ‘good’ end of life care [54]. Therefore, both medicine and society play an important role in deciding what constitutes a ‘good’ end of life. The term ‘*accompagnement*’ finally allows solidarity and autonomy to combine. Even if, in practice, the focus on patient support may lead to shared decision-making, the question at stake is not to reach a shared decision process or some kind of ‘mutuality’ in the patient-physician relationship [60].

If communitarianism is understood as a perspective that places the needs of society over those of the patient, France does not opt for a communitarian approach to end-of-life care when combining solidarity with autonomy. Neither does France opt for a liberal approach that privileges individual autonomy or an intermediary solution such as the ‘responsive communitarianism’ defined by Amitai Etzioni, according to which autonomy and solidarity are part of a conflict that may never be fully eliminated, but can be worked out on a procedural basis [61]. A different pattern is set by the recent shift of the French debate about end-of-life care: autonomy *is not* respected because individual freedom is the prevalent (if not unique) moral and political value, but rather because society as a whole grants importance to individual freedom and wants to care for the dying in the name of solidarity. Here, solidarity is not opposed to autonomy: it rather combines with it, in the sense that autonomy is granted importance to the extent that it is recognised as a value of society as a whole.

We argue that this combination between solidarity and autonomy in the context of the French end-of-life debate is made possible by a conception of the individual as part of the whole, or to put it in Ricoeur’s words, as able to think of ‘oneself as another’ [62]. This conception develops a social (as opposed to an atomistic) understanding of human life and personal identity [12]. Thinking of oneself as existing within the limits of society thus appears as the condition for the recognition of individual freedom. This combination also relates to a conception of solidarity understood as the expression of a shared social duty towards dying and vulnerable patients. This shared social duty is not to be understood in the sense of Rousseau’s general will, but as the expression of a social duty to support each other, namely, to support the weaker members of society.

This conception also entails that granting patient autonomy a central position does not entail the moral legitimacy of every request to die. The increased focus on patient autonomy in French society has its limits when speaking of autonomy as self-determination. For example, the most recent law of 2016 does not allow euthanasia, even in exceptional cases. Patients may make their own decisions, yet they are not allowed to transgress the values of a society that is not ready to define a legal framework for ‘active’ forms of assisted dying.

Thanks to the social conception of the individual, the search for a balance between ‘individual freedom’ and the ‘common interest’ is made possible [54]. Nonetheless, the use of the term ‘*accompagnement*’ should not blind one to the existence of divergent views on what is ‘good’ end-of-life care. These differences cannot be ignored despite the attempt to harmonise autonomy and solidarity, as described in this article. As we have stressed, there is still strong disagreement as to how much autonomy should be granted to a patient at the end of life both when patients are able to express their will and when they are not and have left advanced directives [55].

## Conclusion

Our article shows how the association of the ideas of solidarity and autonomy led to the social acceptance of the latter in the French context. Whereas solidarity is a determining value in French society, at least within the French health care system, respect for patient autonomy has long been considered a foreign concept, deriving from the liberal Anglo-American tradition. At first, respect for patient autonomy was associated with the right to a self-determined death, a request that seemed incompatible with French values, which are based on a model of social cohesion and emphasises the protection of vulnerable members of society such as dying persons. Patient autonomy was only gradually recognised as an important value in the French end-of-life debate, yet to this day, its practical implementation is not evident and its scope remains restricted. It was the redefinition of ‘*accompagnement*’, a notion originally reserved to describe palliative care, that triggered a shift in the debate. This shift happened in 2012 when ‘*accompagnement*’ was employed to justify the right to terminal sedation at a patient’s request. The respect for patient wishes was associated with some kind of social duty towards the dying person, and now appears as being consistent with the general will of society as a whole. It is precisely this association between autonomy and solidarity that allows the leading moral principle of solidarity to define the limits of individual self-determination.

The case of the French end-of-life debate illustrates clearly that solidarity and autonomy are not *per se* incompatible but can inform each other’s meaning and understanding in a particular context. In the French context, this association leads to a definition of autonomy that is promoted as a common social value, on the one hand, and limited by social values such the protection of the dying person, on the other hand. In this definition, solidarity and autonomy become two compatible values, although this compatibility is limited. We see this as an important contribution to the bioethical debate, which has been inclined to emphasise the incompatibility of Anglo-American and Continental European values.

## Footnotes

1 We would like to point out that the French end-of-life debate mainly focuses on cases that take place in a hospital setting.
